# Longitudinal changes in circulating biomarkers from baseline to week 48 in treatment-Naïve people living with HIV initiating integrase inhibitor-based antiretroviral therapy

**DOI:** 10.1371/journal.pone.0343230

**Published:** 2026-02-18

**Authors:** Jose-Ramon Blanco, Miguel Torralba, María Saumoy, Antonia Alcaraz, Mariano Matarranz del Amo, Julian Olalla, Fernando Dronda, Nisa Boukichou-Abdelkader, Enrique Bernal, Sergio Padilla, Joaquim Peraire, Francisca Artigues, María-Jesús Bustinduy-Odriozola, Helena Albendin Iglesias, Alberto Delgado, Arkaitz Imaz, Laura Pérez-Martinez, CoR.I.S. Cohort

**Affiliations:** 1 Hospital Universitario San Pedro, Logroño, Spain; 2 Centro de Investigación Biomédico de La Rioja, Logroño, Spain; 3 Hospital Universitario de Guadalajara. Universidad de Alcalá. IDISCAM (Instituto de investigación de Castilla la Mancha), Toledo, Spain; 4 Hospital Universitario de Bellvitge, L'Hospitalet de Llobregat, Spain; 5 Hospital General Universitario Reina Sofía (Murcia), Murcia, Spain; 6 Hospital Universitario Infanta Leonor, Madrid, Spain; 7 Hospital Costa del Sol, Marbella, Spain; 8 Hospital Universitario Ramón y Cajal, Madrid, Spain; 9 Hospital General Universitario and Universidad Miguel Hernández de Elche. CIBERINFEC, Carlos III Health Institute, Madrid, Spain; 10 Hospital Universitari Joan XXIII de Tarragona, Universitat Rovira i Virgili, Tarragona, CIBERINFEC, Carlos III Health Institute, Tarragona, Spain; 11 Hospital Universitario Son Espases, Illes Balears, Spain; 12 Hospital Universitario Donostia, Gipuzkoa, Spain; 13 Hospital Universitario Virgen de la Arrixaca, El Palmar, Spain; University of California San Diego, UNITED STATES OF AMERICA

## Abstract

**Background:**

Currently, integrase strand-transfer inhibitors (INSTIs) are the cornerstone of antiretroviral therapy (ART), providing potent viral suppression and good tolerability. Emerging evidence suggests that INSTI-based regimens may exert different effects on immune and metabolic pathways, potentially influencing inflammation and comorbidity risk. This study aimed to evaluate the impact of various first-line INSTI-based regimens on a panel of circulating biomarkers in treatment-naïve individuals with HIV.

**Methods:**

We included ART-naïve adults (≥18 years) with confirmed HIV-1 infection initiating a non-boosted INSTI according to the treating physicians’ decisions. The regimen included were bictegravir/emtricitabine/tenofovir alafenamide (BIC/TAF/FTC or Group 1 [G1]), dolutegravir/lamivudine (DTG/3TC or Group 2 [G2]), and dolutegravir/abacavir/lamivudine (DTG/ABC/3TC or Group 3 [G3]). Participants receiving DTG/ABC/3TC were enrolled via the Spanish CoRIS cohort, with samples from the HIV BioBank. Blood samples were collected at baseline and after 48 weeks. Biomarkers were grouped into six categories: pro- and anti-inflammatory cytokines, immune activation, endothelial dysfunction, metabolic markers, and tryptophan catabolism. Changes from baseline were analyzed using Kruskal–Wallis and Dunn’s tests; linear mixed-effects models assessed longitudinal trends.

**Results:**

We included 62 participants. All regimens achieved viral suppression and increased CD4 + counts, with the greatest CD4 + gain in G3. At baseline, G3 had higher TNF, CD40, ICAM-1, and lower IL-10 and leptin levels. After 48 weeks, G3 showed a significant increase in IL-10 and greater declines in CD163 and ICAM-1. Mixed models confirmed distinct longitudinal patterns in CD4 + counts, CD163, and IL-10 in G3.

**Conclusions:**

All INSTI-based regimens led to immune restoration, but modest differences in biomarker trajectories suggest distinct immunomodulatory effects. The clinical relevance of these differences remains unclear and warrants further study to assess their role in long-term comorbidity risk.

## Introduction

The lifelong administration of antiretroviral therapy (ART) requires adherence to treatment strategies that ensure sustained both virological efficacy and long-term safety. However, prolonged ART exposure has been associated with a range of adverse effects [1], underscoring the need for robust evidence to guide optimal regimen selection.

Despite the efficacy of ART in suppressing viral replication, people living with HIV (PLWH) continue to exhibit a higher burden of non-AIDS comorbidities compared to uninfected individuals of similar age [2,3]. Multiple mechanisms contribute to the development of non-HIV-related diseases in PLWH, with persistent immune activation and chronic systemic inflammation playing a central role [4–6]. This inflammatory state may be further amplified by bacterial translocation, underlying comorbidities, and concurrent infections [7]. Circulating biomarkers such as C-reactive protein (CRP), interleukin-6 (IL-6), soluble CD14 (sCD14), soluble CD163 (sCD163), and soluble vascular cell adhesion molecule-1 (sVCAM-1) have been independently associated with all-cause mortality in PLWH [6,8,9]. Moreover, dysregulation of adiponectin levels and alterations in kynurenine pathway metabolites have been linked to an increased risk of non-AIDS-related clinical outcomes [10–12]. Nonetheless, there is currently no consensus regarding the most reliable inflammatory biomarkers for clinical prognostication.

Currently, integrase strand-transfer inhibitors (INSTIs) are a cornerstone of ART, providing potent viral suppression, high tolerability, and a favorable safety profile [13]. Their efficacy has led to their widespread recommendation as first-line therapy in treatment-naïve PLWH [14,15]. Among available INSTIs, dolutegravir (DTG) and bictegravir (BIC) are the most commonly prescribed. Emerging evidence suggests that INSTI-based regimens may differentially modulate immunologic and metabolic pathways [16–18], resulting in distinct profiles of systemic inflammation and immune activation. However, these effects are not consistently observed across all studies [19–21]. A deeper understanding of these differences may have important implications for the long-term risk of comorbidity in PLWH.

Understanding the impact of different initial INSTI-based regimens during effective ART is essential to improving our knowledge of the pathogenesis of HIV-related comorbidities. To address this knowledge gap, we assessed a panel of pathophysiological biomarkers in treatment-naive individuals initiating different INSTI-based regimen. Our objective was to determine whether specific regimens were associated with more favorable biomarker profiles after 48 weeks of therapy, thereby identifying potential advantages in reducing long-term comorbidity risk in PLWH.

## Methods

### Ethical considerations

Initial approval for this study was obtained from the Comité Ético de Investigación de La Rioja (CEImLAR), which acted as the reference ethics committee, and was subsequently obtained from the ethics committees of all participating institutions. All participants provided written informed consent for the collection and storage of blood samples, as well as for the collection of associated personal and clinical data. The CoRIS cohort was approved by the Research Ethics Committee of the Gregorio Marañón Hospital.

### Participants

In this study (March 1, 2021 – June 30, 2024), we recruited ART-naive adults (aged ≥18 years) with confirmed HIV-1 infection. Participants were prescribed a non-boosted INST-regimen according to the treating physicians’ decisions in real-world clinical practice. The regimen included were bictegravir/emtricitabine/tenofovir alafenamide (BIC/TAF/FTC or Group 1 [G1]), dolutegravir/lamivudine (DTG/3TC or Group 2 [G2]), and dolutegravir/abacavir/lamivudine (DTG/ABC/3TC or Group 3 [G3]).

Eligible patients were required to have a baseline CD4 + T-cell count ≥200 cells/mL. Exclusion criteria included hepatitis B or C coinfection, new AIDS-defining conditions within 30 days of screening, pregnancy, and known CVD or diabetes mellitus. These criteria ensured a clinically stable, treatment-naïve population and reduced potential confounding from conditions known to influence circulating biomarkers.

Sample size was determined by the number of eligible participants during the recruitment period, which may limit power to detect modest between-regimen differences. Accordingly, the study was designed as exploratory and hypothesis-generating, with prespecified emphasis on within-person biomarker changes from baseline to w48, and between-regimen comparisons considered secondary.

No participants had documented SARS-CoV-2 infection during the study period.

### Blood samples

Blood samples were collected at baseline and again during a second period, approximately 48 weeks after initiating ART. Serum samples were obtained from blood drawn during a study visit and stored at −80° C. For participants receiving DTG/ABC/3TC regimens, data were obtained from the Spanish HIV-positive cohort (CoRIS), and biological samples were provided by the HIV BioBank integrated within the Spanish AIDS Research Network. Briefly, CoRIS is a multicenter cohort that collects a standardized dataset, including sociodemographic, immunological, and clinical information at baseline and through follow-up [22,23]. This cohort is linked to a centralized biorepository, the Spanish HIV Hospital Universitario Gregorio Marañón Biobank (HIV HUGM BioBank) [24]. Participating centers follow standardized protocols to collect an initial blood sample prior to ART initiation and, when possible, additional samples annually during follow-up. All participants provided specific informed consent for the storage and use of their biological samples in the BioBank. To ensure confidentiality and compliance with ethical standards, the authors had no access to any personally identifiable information about the participants.

### Circulating biomarkers

We assessed a comprehensive panel of circulating biomarkers, categorized into six categories: a) pro-inflammatory cytokines: C-reactive protein (CRP), tumor necrosis factor (TNF), interleukin (IL)-6, and IL-8; b) anti-inflammatory cytokines, represented by IL-10; c) immune activation markers: (sCD163), and lipopolysaccharide-binding protein (LBP); d) endothelial and vascular dysfunction markers: intracellular adhesion molecule-1 (ICAM-1), vascular cell adhesion molecule-1 (VCAM-1), E-selectin, P-selectin, and soluble P-selectin; e) metabolic and adipokine markers: leptin and adiponectin; and f) tryptophan catabolism markers: tryptophan, kynurenic acid, and quinolinic acid.

CD14, CD40, LBP, TNF, sCD163, ICAM-1, IL-6, IL-8, IL-10, E-selectin, P-selectin, VCAM-1 and adiponectin were measured using human magnetic Luminex assays (BioTechne, Minneapolis, USA; catalogue LXSAHM), following the manufacturer´s instructions. CRP, leptin, adiponectin, tryptophan, kynurenic acid, and quinolinic acid were quantified by an enzyme-linked immunosorbent assay kit (ELISA) according to standard protocols. Assay sensitivity and minimum detectable dose (MDD) are detailed in S1 Table.

This biomarkers panel was deliberately selected to capture complementary and interrelated pathways –chronic inflammation, immune activation, microbial translocation, endothelial dysfunction, metabolic dysregulation, and altered tryptophan metabolism– implicated in HIV pathogenesis and cardiometabolic comorbidities [6,8–12,25–30].

### Statistical analysis

Descriptive statistics for baseline quantitative variables were summarized using medians and interquartile ranges (IQRs) due to non-normal distributions assessed by the Shapiro-Wilk test. Categorical variables were described using counts and percentages. For baseline comparisons between treatment groups, the Kruskal-Wallis test was used for continuous variables and the chi-square or Fisher’s exact test for categorical variables, as appropriate.

Missing data for “smoker”, “alcohol consumption frequency”, and “body mass index (BMI)” were imputed using multiple imputation by chained equations (MICE) [31], treating variables as ordered categorical (ordinal logistic regression; method = “polr”). Five imputed datasets were generated (m = 5, seed = 123) and estimates were pooled across imputations following Rubin’s rules.

Absolute changes from baseline to week 48 (w48) (Δ = w48 − baseline) were summarized using medians and IQRs and compared across groups using Kruskal-Wallis tests; when the overall test was significant (p < 0.05), post hoc pairwise comparisons used Dunn’s test with Holm adjustment. Effect sizes for Kruskal-Wallis tests were summarized using epsilon-squared (ε²). Within-group changes from baseline to w48 were additionally assessed using paired Wilcoxon signed-rank tests. Within-group changes from baseline to w48 were described using paired Wilcoxon signed-rank tests, whereas formal longitudinal inference relied on the main time effect and time-by-group interaction terms in the linear mixed-effects models.

Longitudinal changes were analyzed using linear mixed-effects models with random intercepts for each participant. Fixed effects included time (baseline vs w48), treatment group (with G1 as reference), and a time-by-group interaction, adjusting for sex and alcohol consumption, which differed at baseline between groups. Results are reported as regression coefficients (β) with 95% confidence intervals.

All statistical tests were two-sided. For post hoc comparisons, Holm-adjusted p values are reported; otherwise, p values are nominal. Given the exploratory nature and multiple biomarkers, results were interpreted with emphasis on effect sizes and confidence intervals rather than statistical significance alone. Analyses were conducted using R software [32] and relevant packages for data manipulation and modeling [33–36].

## Results

### Participant characteristics at baseline

A total of 62 naïve PLWH were included. Baseline characteristics by study group are shown in Table 1. Between-group differences were observed in sex distribution, with men predominating across all groups and reaching 100% in G3 (p = 0.013), and in alcohol consumption, with low or no alcohol use less frequent in G3 (p = 0.003). No significant between-group differences were observed in HIV-related parameters.

**Table 1:** Baseline characteristics by the study groups.

Variable	Group 1 (n = 20)	Group 2 (n = 20)	Group 3 (n = 22)	p-value
Age (years)	33.3 [24.2-38.1]	33.1 [28.5-40.0]	36.2 [30.7-44.4]	0.320
Male (n(%))	15 (75%)	19 (95%)	22 (100%)	**0.013**
Spanish (n(%))	10 (50%)	12 (60%)	15 (68.2%)	0.487
University degree (n(%))	10 (50%)	12 (60%)	11 (50%)	0.762
Never smoked (n(%))	11 (55%)	11 (55%)	11 (50%)	0.931
Low or no alcohol consumption n(%))	16 (80%)	17 (85%)	9 (40.9%)	**0.003**
BMI	25.1 [21.8-30.6]	22.6 [21.7-24.9]	24.9 [23.1-26.4]	0.203
**Related to HIV infection**				
Sexual HIV transmission (n(%))	18 (90%)	18 (90%)	22 (100%)	0.377
CD4 (cells/mL)	421 [349-523]	536 [419-682]	414 [313-612]	0.281
Viral load (copies/ml)	26762 [7072.5-107044.25]	13058 [7150-37500]	31721 [16775-75808]	0.146

**Note:** Group comparisons were performed using the Chi-square or Fisher’s exact test, as appropriate. Quantitative variables are expressed as median [interquartile range]. Non-parametric comparisons were performed using the Kruskal–Wallis test.

**Abbreviations:** BMI, body mass index

### Baseline biomarker differences across regimens

Baseline biomarker levels were compared across groups using non-parametric tests (Table 2). Several inflammatory, immune activation, endothelial, adipokine, and tryptophan metabolism markers differed significantly between groups. Among pro- and anti-inflammatory biomarkers, TNF and IL-10 showed significant between-group differences, with higher TNF levels and markedly lower IL-10 concentrations in G3 compared with G1 and G2. For immune activation markers, CD40 differed significantly across groups, driven by higher levels in G3, whereas CD163, CD14, and LBP showed no significant baseline differences. Regarding endothelial and vascular dysfunction markers, ICAM-1 and soluble P-selectin differed significantly between groups, with higher ICAM-1 and lower soluble P-selectin levels observed in G3. In the adipokine profile, leptin levels were significantly lower in G3 compared with both G1 and G2, while adiponectin did not differ between groups. Markers of tryptophan metabolism showed pronounced baseline differences: tryptophan, kynurenic acid, and the KA/QA ratio were significantly lower in G3, whereas quinolinic acid levels were comparable across groups.

**Table 2:** Baseline biomarker levels by study group.

Biomarker	Group 1 (n = 20)	Group 2 (n = 20)	Group 3 (n = 22)	p-value (global)	Post hoc comparisons
**Pro- and anti-inflammatory biomarkers**					
CRP (ng/ml)	1490.82 [1172.28–1947.49]	1145.41 [733.32–1716.03]	1222.21 [836.1–1758.74]	0.312	
TNF (fg/ml)	34.42 [32.58–40.09]	30.83 [25.98–35.56]	42.47 [37.62–48.82]	**<0.001**	G1 vs. G3 p 0.040 G2 vs. G3 p <0.001
IL-6 (fg/ml)	41.02 [35.9–44.22]	34.94 [28.26–40.23]	39.46 [33.39–43.82]	0.114	
IL-8 (fg/ml)	25.16 [21.22–27.76]	23.33 [17.7–26.69]	26.86 [23.41–29.43]	0.130	
IL-10 (fg/ml)	33.69 [30.76–37.82]	26.65 [23.12–34.7]	12.75 [11.47–14.82]	**<0.001**	G1 vs. G3 p <0.01 G2 vs. G3 p <0.001
**Immune activation biomarkers**					
CD40 (ng/ml)	3.56 [2.33–3.79]	2.53 [1.9–3.73]	4.23 [3.31–5.64]	**0.003**	G1 vs. G3 p 0.05 G2 vs. G3 p 0.003
CD163 (ng/ml)	760.58 [605.02–1051.05]	594.05 [402.12–786.85]	767.37 [590.22–1545.26]	0.093	
CD14 (ng/ml)	2.41 [2.19–2.92]	2.44 [2.06–2.66]	2.62 [2.29–2.94]	0.235	
LBP (ng/ml)	14.16 [11.87–17.62]	12.36 [11–15.43]	13.76 [10.69–18.34]	0.544	
**Endothelial and vascular dysfunction markers**					
ICAM-1 (ng/ml)	49.24 [40.67–63.96]	44.99 [39.13–57.57]	65.03 [59.35–82.53]	**0.041**	G2 vs. G3 p 0.043
VCAM-1 (ng/ml)	925.61 [704.86–1299.45]	744.63 [614.55–1007.6]	880.68 [712.08–1056.22]	0.131	
E-selectin (ng/ml)	32.34 [24.94–36.08]	20.3 [14.68–32.31]	31.51 [26.35–38.03]	0.062	
P-selectin (ng/ml)	30.94 [26.2–36.28]	27.84 [18.32–33.1]	31.61 [25.79–34.41]	0.336	
Soluble P-selectin (ng/ml)	57.41 [48.13–74.55]	55.77 [37.91–69.15]	42.48 [33.1–55.66]	**0.046**	-
**Adipokine markers**					
Leptin (ng/ml)	4.43 [0.97–11.72]	2.12 [1.43–4.31]	0.06 [0.05–0.09]	**<0.001**	G1 vs. G3 p <0.001 G2 vs. G3 p <0.001
Adiponectin (ng/ml)	9.91 [8.2–11.58]	9.06 [7.6–11.94]	10.65 [8.38–13.65]	0.244	
Tryptophan metabolism					
Triptofan (ng/ml)	4.73 [3.08–10.01]	3.44 [3.15–3.79]	2.14 [1.62–2.49]	**<0.001**	G1 vs. G3 p <0.001 G2 vs. G3 p <0.001
Kynurenine acid (ng/ml)	45.55 [40.72–48.76]	48.34 [42.89–51.92]	28.2 [25.42–32.37]	**<0.001**	G1 vs. G3 p <0.001 G2 vs. G3 p <0.001
Quinolinic acid (ng/ml)	8.73 [8.32–10.21]	9.32 [8.59–11.54]	8.65 [7.69–9.71]	0.261	
KA/QA ratio	5.17 [4.49–5.65]	4.77 [4.12–5.66]	3.32 [2.74–3.86]	**<0.001**	G1 vs. G3 p <0.001 G2 vs. G3 p <0.001

**Note:** Values are presented as median [interquartile range]. Global between-group comparisons were performed using the Kruskal–Wallis test. When the overall test was significant (p < 0.05), post hoc pairwise comparisons were conducted using Dunn’s test with Holm correction. Only Holm-adjusted p values are reported. **Abbreviations:** CD14, cluster of differentiation 14; CD40, cluster of differentiation 40; CD163, cluster of differentiation 163; CRP, C-reactive protein; ICAM-1, intercellular adhesion molecule 1; IL, interleukin; KA/QA ratio, kynurenic acid to quinolinic acid ratio; LBP, lipopolysaccharide-binding protein; TNF, tumor necrosis factor; VCAM-1, vascular cell adhesion molecule 1.

Post hoc pairwise comparisons using Dunn’s test with Holm correction indicated that the significant global effects for TNF, IL-10, CD40, ICAM-1, leptin, tryptophan, kynurenic acid, and the kynurenic acid-to-quinolinic acid (KA/QA) ratio were mainly driven by differences involving G3 vs. G1 and/or G2, whereas G1 vs. G2 comparisons were not significant after correction (Table 2). Although soluble P-selectin showed a significant overall Kruskal–Wallis test, none of the pairwise comparisons remained significant after adjustment.

### Within-group changes from baseline to w48

Within-group analyses showed a significant increase in CD4 ⁺ T-cell count (cells/mL) from baseline to w48 in all groups (Table 3). Median changes were +143.5 (60–249.5) in G1 (p < 0.001), + 270 (152.5–545) in G2 (p < 0.001), and +331.5 (222–372.75) in G3 (p < 0.001), with the largest median increase observed in G3. All participants achieved an undetectable HIV viral load by w48. Beyond CD4 + recovery, most biomarkers showed limited within-group change over the 48-week follow-up. Among pro- and anti-inflammatory markers, IL-10 increased significantly in G3 (p < 0.001), while CRP, TNF, IL-6 and IL-8 did not change significantly in any group. For immune activation markers, CD163 decreased significantly in G3 (p < 0.001), whereas CD40, CD14 and LBP remained stable. Among endothelial and vascular dysfunction markers, ICAM-1 decreased in G2 (p = 0.038) and G3 (p = 0.002), and VCAM-1 decreased in G1 (p = 0.001) and G3 (p < 0.001). E-selectin decreased in G3 (p = 0.014), while P-selectin and soluble P-selectin showed no significant within-group changes. No significant within-group changes were detected for leptin or adiponectin, and tryptophan metabolism markers were largely stable, except for a modest reduction in the KA/QA ratio in G1 (p = 0.019).

**Table 3:** Within-group changes in biomarker levels from baseline to w48.

Biomarker	Group	Basal	w48	Δ (w48-basal)	p-value
**Pro- and anti-inflammatory biomarkers**					
CRP (ng/ml)	G1	1490.82 [1172.28–1947.49]	1790.12 [1337.07–2045.41]	126.67 [-305.21–574.79]	0.475
	G2	1145.41 [733.32–1716.03]	1284.16 [879.36–1829.99]	121.25 [-444.38–398.96]	1.000
	G3	1222.21 [836.1–1758.74]	1152.7 [752.76–1441.65]	-120.69 [-589.03–329.58]	0.406
TNF (fg/ml)	G1	34.42 [32.58–40.09]	33.28 [29.86–37.54]	-1.94 [-4.48–2.65]	0.277
	G2	30.83 [25.98–35.56]	30.91 [28.29–33.76]	-2.8 [-6.33–4.19]	0.571
	G3	42.47 [37.62–48.82]	37.57 [35.77–40.34]	-4.57 [-12.56–2.84]	0.061
IL-6 (fg/ml)	G1	41.02 [35.9–44.22]	38.36 [33.94–45.09]	0.75 [-4.83–6.28]	0.841
	G2	34.94 [28.26–40.23]	35.47 [31.65–38.9]	-2 [-4.94–5.83]	0.898
	G3	39.46 [33.39–43.82]	37.15 [34.09–41.69]	0.09 [-8.16–2.18]	0.532
IL-8 (fg/ml)	G1	25.16 [21.22–27.76]	24.51 [22.2–26.28]	-0.06 [-3.43–2.57]	0.955
	G2	23.33 [17.7–26.69]	23.66 [20.63–27.4]	-0.71 [-4.53–5.8]	0.812
	G3	26.86 [23.41–29.43]	25.8 [23.25–31.01]	-0.08 [-3.49–4.78]	0.922
IL-10 (fg/ml) (fg/ml)	G1	33.69 [30.76–37.82]	30.11 [27.39–36.34]	-2.08 [-3.91–1.82]	0.365
	G2	26.65 [23.12–34.7]	30.32 [24.22–35.23]	0.46 [-2.87–5.76]	0.387
	G3	12.75 [11.47–14.82]	30.54 [28.89–35.55]	18.96 [15.23–24.04]	**<0.001**
**Immune activation biomarkers**					
CD40 (ng/ml)	G1	3.56 [2.33–3.79]	2.62 [2.22–3.85]	-0.16 [-0.68–0.43]	0.622
	G2	2.53 [1.9–3.73]	2.69 [2.02–3.18]	0 [-0.84–0.87]	0.869
	G3	4.23 [3.31–5.64]	4.61 [3.37–5.45]	-0.09 [-0.65–0.81]	0.889
CD163 (ng/ml)	G1	760.58 [605.02–1051.05]	743.82 [548.42–917.25]	-34.86 [-315.28–128.01]	0.294
	G2	594.05 [402.12–786.85]	513.41 [353.28–840.12]	-91.45 [-267.1–129.96]	0.133
	G3	767.37 [590.22–1545.26]	525.29 [386.95–695.32]	-366.4 [-679.29–-200.14]	**<0.001**
CD14 (ng/ml)	G1	2.41 [2.19–2.92]	2.44 [2.2–2.85]	0.28 [-0.15–0.4]	0.202
	G2	2.44 [2.06–2.66]	2.27 [2.12–2.61]	-0.01 [-0.35–0.39]	0.985
	G3	2.62 [2.29–2.94]	2.35 [2.14–2.99]	-0.15 [-0.82–0.33]	0.406
LBP (ng/ml)	G1	14.16 [11.87–17.62]	13.95 [12.07–16.28]	0.43 [-2.63–2.25]	0.927
	G2	12.36 [11–15.43]	12.22 [11.39–15.67]	0.4 [-1.26–4.61]	0.261
	G3	13.76 [10.69–18.34]	13.98 [10.18–17.17]	-0.63 [-2.48–3.36]	0.975
**Endothelial and vascular dysfunction markers**					
ICAM-1(ng/ml)	G1	49.24 [40.67–63.96]	50.45 [41.44–57.57]	-4.46 [-6.67–0.49]	0.114
	G2	44.99 [39.13–57.57]	41.73 [35.65–56.23]	-4.93 [-8.62–0.64]	**0.038**
	G3	65.03 [59.35–82.53]	60.3 [54.87–72.31]	-9.52 [-13.53–-1.84]	**0.002**
VCAM-1 (ng/ml)	G1	925.61 [704.86–1299.45]	775.01 [676.84–853.28]	-189.44 [-490.91–-16.27]	**0.001**
	G2	744.63 [614.55–1007.6]	569.29 [493.51–690.76]	-207.37 [-341.16–-35.59]	0.123
	G3	880.68 [712.08–1056.22]	545.61 [446.03–688.92]	-330.13 [-553–-201.27]	**<0.001**
E-selectin (ng/ml)	G1	32.34 [24.94–36.08]	24.62 [22.57–44.4]	1.81 [-6.74–9.85]	0.648
	G2	20.3 [14.68–32.31]	22.98 [15.05–34.55]	-1.34 [-6.73–12.09]	0.927
	G3	31.51 [26.35–38.03]	29.09 [19.74–33.52]	-6.64 [-12.38–-0.05]	**0.014**
P-selectin (ng/ml)	G1	30.94 [26.2–36.28]	28.46 [24.67–34.46]	0.78 [-5.72–5.93]	1.000
	G2	27.84 [18.32–33.1]	25.17 [23.58–32.36]	-1.98 [-6.8–6.97]	0.784
	G3	31.61 [25.79–34.41]	29.06 [24.89–33.96]	-0.95 [-6.64–2.47]	0.463
Soluble P-selectin (ng/ml)	G1	57.41 [48.13–74.55]	57.07 [46.37–71.05]	-1.49 [-12.3–10.92]	0.985
	G2	55.77 [37.91–69.15]	53.89 [43.26–67.65]	-2.7 [-15.16–16.68]	0.841
	G3	42.48 [33.1–55.66]	47.01 [37.63–58.98]	5.4 [-5.44–15.62]	0.222
**Adipokine markers**					
Leptin (ng/ml)	G1	4.43 [0.97–11.72]	8.03 [2.68–17.01]	1.88 [-0.2–7.56]	0.07
	G2	2.12 [1.43–4.31]	2.4 [1.75–5.22]	0.5 [-0.02–1.7]	0.056
	G3	0.06 [0.05–0.09]	0.07 [0.06–0.15]	0.01 [0–0.05]	0.055
Adiponectin (ng/ml)	G1	9.91 [8.2–11.58]	10.56 [7.09–14.16]	-0.09 [-2.44–1.57]	1.000
	G2	9.06 [7.6–11.94]	10.41 [8.21–12.59]	0.15 [-1.01–1.08]	0.756
	G3	10.65 [8.38–13.65]	11.46 [9.43–14.61]	0.99 [-1.25–1.85]	0.305
**Tryptophan metabolism**					
Tryptophan (ng/ml)	G1	4.73 [3.08–10.01]	3.78 [3–7.15]	0.06 [-2.32–4.14]	0.546
	G2	3.44 [3.15–3.79]	3.02 [2.76–4.9]	-0.47 [-0.64–1.82]	0.927
	G3	2.14 [1.62–2.49]	2.04 [1.44–2.61]	-0.01 [-0.24–0.24]	0.835
Kynurenine acid (ng/ml)	G1	45.55 [40.72–48.76]	41.59 [38.63–47.7]	-1.15 [-5.18–0.37]	0.123
	G2	48.34 [42.89–51.92]	47.42 [43.11–50.3]	-3.74 [-5.09–4.06]	0.294
	G3	28.2 [25.42–32.37]	27.55 [24.39–31.99]	-0.86 [-5.68–2.72]	0.388
Quinolinic acid (ng/ml)	G1	8.73 [8.32–10.21]	9.98 [8.15–10.86]	0.55 [-0.55–1.53]	0.189
	G2	9.32 [8.59–11.54]	8.94 [7.66–12.08]	0.36 [-1.1–1.53]	0.701
	G3	8.65 [7.69–9.71]	8.61 [7.59–10.05]	-0.19 [-0.59–0.73]	0.899
KA/QA ratio	G1	5.17 [4.49–5.65]	4.45 [3.97–4.99]	-0.56 [-1.22–-0.01]	**0.019**
	G2	4.77 [4.12–5.66]	4.62 [3.5–5.86]	-0.23 [-0.8–0.09]	0.216
	G3	3.32 [2.74–3.86]	3.2 [2.65–3.68]	-0.24 [-0.89–0.56]	0.210

**Note:** Values are presented as median [interquartile range]. Within-group comparisons between baseline and w48 were performed using the paired Wilcoxon signed-rank test. Δ represents the median of individual changes (w48 − baseline). P values refer to within-group comparisons. **Abbreviations:** CD14, cluster of differentiation 14; CD40, cluster of differentiation 40; CD163, cluster of differentiation 163; CRP, C-reactive protein; G, group; ICAM-1, intercellular adhesion molecule 1; IL, interleukin; KA/QA ratio, kynurenic acid to quinolinic acid ratio; LBP, lipopolysaccharide-binding protein; TNF, tumor necrosis factor; VCAM-1, vascular cell adhesion molecule 1.

### Between-group differences in changes from baseline to w48

Between-group comparisons of CD4 + T-cell recovery (Δ w48 − baseline) showed a significant difference across groups (Kruskal–Wallis p = 0.013; ε² = 0.113). Dunn–Holm post hoc testing indicated greater CD4 + gains in G2 and G3 compared with G1, whereas changes did not differ between G2 and G3. For other biomarkers, significant between-group differences in within-group changes were observed only for IL-10 and CD163 (**Table 4**). IL-10 exhibited a strong group effect (p < 0.001; ε² = 0.513), driven by a marked increase in G3 relative to both G1 and G2 (Dunn–Holm p < 0.001 for both). Similarly, changes in CD163 differed across groups (p = 0.002; ε² = 0.174), with a greater reduction in G3 compared with G1 and G2. No significant between-group differences in Δ were detected for the remaining pro- and anti-inflammatory markers, endothelial and vascular dysfunction markers, adipokines, or tryptophan metabolism markers (all p ≥ 0.056).

**Table 4:** Between-group comparison of within-group changes (Δ) from baseline to w48.

Biomarker	Δ Group 1 (n = 20)	Δ Group 2 (n = 20)	Δ Group 3 (n = 22)	P KW on (Δ)	Post hoc (Dunn–Holm, adjusted p)	ε²
**Pro- and anti-inflammatory biomarkers**						
CRP (ng/ml)	126.67 [-305.21-574.79]	121.25 [-444.38–398.96]	-120.69 [-589.03-329.58]	0.563	—	-0.014
TNF (fg/ml)	-1.94 [-4.48–2.65]	-2.8 [-6.33–4.19]	-4.57 [-12.56–2.84]	0.472	—	-0.008
IL-6 (fg/ml)	0.75 [-4.83–6.28]	-2 [-4.94–5.83]	0.09 [-8.16–2.18]	0.822	—	-0.027
IL-8 (fg/ml)	-0.06 [-3.43–2.57]	-0.71 [-4.53–5.8]	-0.08 [-3.49–4.78]	0.996	—	-0.034
IL-10 (fg/ml)	-2.08 [-3.91–1.82]	0.46 [-2.87–5.76]	18.96 [15.23–24.04]	**<0.001**	G1 vs. G3: <0.001 G2 vs. G3: <0.001	0.513
**Immune activation biomarkers**						
CD40 (ng/ml)	-0.16 [-0.68–0.43]	0 [-0.84–0.87]	-0.09 [-0.65–0.81]	0.974	—	-0.033
CD163 (ng/ml)	-34.86 [-315.28–128.01]	-91.45 [-267.1–129.96]	-366.4 [-679.29–-200.14]	**0.002**	G1 vs. G3 0.009 G2 vs. G3 0.005	0.174
CD14 (ng/ml)	0.28 [-0.15–0.4]	-0.01 [-0.35–0.39]	-0.15 [-0.82–0.33]	0.457	—	-0.007
LBP (ng/ml)	0.43 [-2.63–2.25]	0.4 [-1.26–4.61]	-0.63 [-2.48–3.36]	0.721	—	-0.023
**Endothelial and vascular dysfunction markers**						
ICAM-1(ng/ml)	-4.46 [-6.67–0.49]	-4.93 [-8.62–0.64]	-9.52 [-13.53–-1.84]	0.059	—	0.062
VCAM-1 (ng/ml)	-189.44 [-490.91–-16.27]	-207.37 [-341.16–-35.59]	-330.13 [-553–-201.27]	0.131	—	0.035
E-selectin (ng/ml)	1.81 [-6.74–9.85]	-1.34 [-6.73–12.09]	-6.64 [-12.38–-0.05]	0.056	—	0.062
P-selectin (ng/ml)	0.78 [-5.72–5.93]	-1.98 [-6.8–6.97]	-0.95 [-6.64–2.47]	0.916	—	-0.031
Soluble P-selectin (ng/ml)	-1.49 [-12.3–10.92]	-2.7 [-15.16–16.68]	5.4 [-5.44–15.62]	0.572	—	-0.015
**Adipokine markers**						
Leptin (ng/ml)	1.88 [-0.2–7.56]	0.5 [-0.02–1.7]	0.01 [0–0.05]	0.059	—	0.062
Adiponectin (ng/ml)	-0.09 [-2.44–1.57]	0.15 [-1.01–1.08]	0.99 [-1.25–1.85]	0.824	—	-0.027
**Tryptophan metabolism**						
Tryptophan (ng/ml)	0.06 [-2.32–4.14]	-0.47 [-0.64–1.82]	-0.01 [-0.24–0.24]	0.918	—	-0.031
Kynurenine acid (ng/ml)	-1.15 [-5.18–0.37]	-3.74 [-5.09–4.06]	-0.86 [-5.68–2.72]	0.910	—	-0.031
Quinolinic acid (ng/ml)	0.55 [-0.55–1.53]	0.36 [-1.1–1.53]	-0.19 [-0.59–0.73]	0.605	—	-0.017
Kynurenine acid to Quinolinic acid ratio	-0.56 [-1.22–-0.01]	-0.23 [-0.8–0.09]	-0.24 [-0.89–0.56]	0.353	—	0.001

**Note:** Values are presented as median [interquartile range] for Δ (w48 − baseline). Between-group differences in Δ were assessed using the Kruskal–Wallis test. When the overall test was significant (p < 0.05), post hoc pairwise comparisons were performed using Dunn’s test with Holm correction. ε² denotes the effect size for the Kruskal–Wallis test. **Abbreviations:** CD14, cluster of differentiation 14; CD40, cluster of differentiation 40; CD163, cluster of differentiation 163; CRP, C-reactive protein; ICAM-1, intercellular adhesion molecule 1; IL, interleukin; KA/QA ratio, kynurenic acid to quinolinic acid ratio; LBP, lipopolysaccharide-binding protein; TNF, tumor necrosis factor; VCAM-1, vascular cell adhesion molecule 1.

### Longitudinal biomarker trajectories in mixed-effects models

Longitudinal changes were evaluated using linear mixed-effects models adjusted for sex, smoking status, and alcohol consumption, with G1 as the reference group. Models included fixed effects for time (baseline vs w48), group (G2 and G3 vs G1), and their interaction. Significant overall time × group effects (global interaction test) are summarised in Table 5.

**Table 5:** Significant time-by-group Interactions in linear mixed-effects models.

Biomarker	Interaction	β [IC95%]	p(term)	p(global Time×Group)
CD4 (cells/mL)	w48 × G2 (vs G1)	157.70 [31.26–284.14]	0.015	**0.028**
	w48 × G3 (vs G1)	140.70 [16.73–264.67]	0.027	
CD163 (ng/ml)	w48 × G3 (vs G1)	−459.18 [−740.16–-178.21]	0.002	**0.001**
	w48 × G2 (vs G1)	45.20 [−241.46–331.87]	0.753	
IL-10 (fg/ml)	w48 × G3 (vs G1)	16.85 [10.69–23.00]	<0.001	**<0.001**
	w48 × G2 (vs G1)	0.55 [−5.75–6.85]	0.862	

**Note:** Linear mixed-effects models were used to assess time (baseline vs w48), group, and time-by-group interactions for each biomarker, with G1 as the reference group. Models were adjusted for sex and alcohol consumption.

A significant time × group interaction was observed for CD4 ⁺ T-cell count (p(global) = 0.028), with both G2 (p(term) = 0.015) and G3 (p(term) = 0.027) differing from G1 in their w48 change. For CD163, the overall interaction was significant (p(global) 0.001), driven by G3 (p(term) 0.002) rather than G2. IL-10 also showed a significant overall interaction (p(global) <0.001), with a marked divergence in G3 (p(term) <0.001) compared with G1, while no interaction was detected for G2.

## Discussion

In this prospective, non-randomized study of ART-naive individuals initiating INSTI-based regimens who successfully achieved virologic suppression, we observed differences in serum levels of some biomarkers across treatment groups. These findings suggest that inflammation in PLWH is a complex and multifactorial process, potentially modulated by the specific INSTIs regimen employed. To our knowledge, this is the first study to compare longitudinal inflammatory biomarker profiles across three commonly used INSTI-based first-line regimens in treatment-naïve PLWH. Our analyses focused on biomarkers previously linked to serious clinical outcomes [6,8–12,25–30].

Notably, absolute changes in quantitative biomarkers from baseline and w48 showed no significant differences among treatment groups in markers of endothelial and vascular dysfunction, adipokines, or tryptophan metabolism. These findings could support the hypothesis of a potential class effect, whereby these agents exert similar immunologic and inflammatory modulation. These observations are clinically relevant [37], as it suggests that the choice of INSTI may be guided by other therapeutic considerations, without a substantial differential impact on short-term systemic inflammation. In line with these findings, Bailon et al. [38] reported comparable levels of inflammatory markers (including sCD14, fatty acid–binding protein 2, tumor necrosis factor–related apoptosis inducing ligand, interferon-γ–induced protein 10, IL-6, CRP and D-dimer) and similar frequencies of activated and exhausted in ART-naïve PLWH treated with DTG/3TC or DTG/FTC/TAF. Similarly, a longitudinal analysis of cryopreserved samples from treatment-naïve individuals receiving BIC/FTC/TAF, DTG/ABC/3TC, or DTG/FTC/TAF found no significant differences in inflammatory biomarkers over time [20]; D-dimer, sCD14, and TNFR1 declined, while hsCRP and IL-6 remained stable [20]. At 12 months, Calza et al. [39] also observed similar reductions in hsCRP, IL-8, and TNF between BIC/FTC/TAF and DTG/3TC. In contrast, our study identified significant differences suggesting that biomarker responses may vary according to the INST-based regimen employed, particularly in G3. In comparison, G1 and G2 exhibited comparable outcomes, with no statistically significant differences observed between them.

Evidence from multiple studies suggest comparable CD4 + T-cell recovery, across INSTIs-based regimens. In the prospective CoRIS cohort, Suárez-García et al. [40] found no significant differences at w48 among ART-naïve PLWH treated with BIC/FTC/TAF (245 cells/mL), DTG/3TC (259 cells/mL), ABC/3TC/DTG (265 cells/mL). Bailon et al. [38], reported similar findings for DTG/3TC (241 cells/mL) and DTG/FTC/TAF (196 cells/mL). Gallant et al. [41] also found no difference between BIC/TAF/FTC (233 cells/mL) and DTG/ABC/3TC (229 cells/mL). A retrospective study [42] comparing DTG/3TC and BIC/FTC/TAF showed sustained CD4 + T-cell gains with no differences through w84. Quiros-Roldan et al. [43] observed improvements with different regimens including protease inhibitors and INSTIs (raltegravir [RAL], elvitegravir [EVG], and DTG), with only DTG showing significantly greater CD4 + T-cell count increases over time. Similarly, Gan et al. [17] found no significant differences at w48 between DTG/3TC (117 cells/mL) and BIC/FTC/TAF (102 cells/mL). No differences were observed in the study by Calza et al. [39] either. In our cohort, median CD4 + T-cell gains were unexpectedly low in G1 (143 cells/mL) and higher in G3 (331 cells/mL) compared to literature. Gains were +270 cells/mL in G2, with both G2 and G3 showing statistically significant differences versus G1. We also observed an increase in CD4 + T-cell counts over time, with group-specific trajectories in G2 and G3 relative to G1. Although we did not assess CD4/CD8 ratio dynamics in the present study, prior observational data in treatment-naïve individuals initiating ART have shown no differences in CD4/CD8 ratio normalization at w48 between DTG/3TC dual therapy and INSTI-based triple regimens [44], supporting comparable immunologic recovery. We acknowledge the lack of longitudinal CD8 measurements and CD4/CD8 ratio dynamics as an additional limitation, which should be addressed in future prospective studies. These patterns may reflect differential immune reconstitution dynamics, potentially influenced by treatment response or baseline immune status.

The increase in IL-10, a key immunoregulatory cytokine with potent anti-inflammatory properties and a critical role in modulating immune responses and limiting the development of inflammatory and autoimmune disorders [45], observed in G3 may reflect a compensatory anti-inflammatory response. Jianu et al. [46], reported a non-significant reduction in IL-10 levels in response to DTG-based treatment, highlighting the variability in IL-10 dynamics among different regimens. Similarly, Quiros-Roldan et al. [43] observed that one year of effective ART led to significant changes in multiple inflammatory biomarkers, including TNF, sCD14, hs-CRP, IL-7, D-dimer, sCD163, IL-6, and IL-10, although only TNF, sCD14, hs-CRP, IL-7, and sCD163 showed differential values between treatment groups. At 12 months, significant reductions were reported for TNF with DTG, sCD14 with RAL and DTG, D-dimer with all INSTIs, and sCD163 with RAL. In contrast, IL-6 and IL-7 increased significantly with EVG. In our case, IL-10 levels showed a highly significant time × group interaction in G3, despite the absence of a main time effect. This suggests that anti-inflammatory regulation may follow a distinct trajectory in G3, possibly influenced by specific clinical or immunological conditions unique to this group.

In relation to sCD163, this is a marker of macrophage activation involved in modulating inflammatory responses [47]. It plays a protective role by attenuating monocyte hyperactivation during infectious and inflammatory states, in part through the downregulation of pro-inflammatory cytokines such as TNF, IL-1β, IL-6, and IL-8 [47,48]. Additionally, sCD163 may suppress the production of pro-inflammatory chemokines like MCP-1 promote the release of anti-inflammatory mediators such as IL-10 [49], and limited the expression of pro-apoptotic transcription factors [48]. Clinically, however, has been associated with chronic immune activation and adverse outcomes in various inflammatory and infectious conditions, including HIV [47]. Regarding ART, Quirós-Roldán et al. [43] reported a significant decline in SCD163 with RAL but not with DTG. Similarly, Beltrán et al. [50] observed that sCD163 levels decreased after 48 weeks of ART, although levels remained higher than in HIV-negative controls. Notably, those receiving protease inhibitors had a smaller reduction in sCD163 levels than those on PI-sparing regimens. Importantly, no participants in that cohort received INSTIs, in contrast to our study. In our cohort, sCD163 levels decreased from baseline to w48, with the largest reduction observed in G3. In mixed-effects models, a significant time × group interaction indicated a distinct temporal profile in G3 relative to G1, consistent with group-specific modulation of monocyte/macrophage activation. These findings suggest differential inflammatory resolution across regimens, although mechanistic inferences should be made cautiously.

The simultaneous and significant modulation of IL-10 and sCD163 observed in G3 may reflect two non-mutually exclusive phenomena. This pattern may reflect activation of anti-inflammatory pathways and a shift toward an immunoregulatory phenotype, consistent with M2 macrophage polarization and IL-10-mediated immune modulation [51]. Alternatively, these changes could represent a compensatory response to immune activation or endothelial dysfunction, with IL-10 and sCD163 serving to limit inflammatory damage [52]. The magnitude and clinical relevance of these changes require confirmation in prospective studies with extended follow-up and validated endpoints.

### Strengths

A key strength of this study is the prospective, real-world longitudinal profiling of a broad inflammatory biomarker panel from ART initiation to w48 across contemporary INSTI-based regimens under standardized procedures.

### Limitations

This study has several limitations. First, the relatively small sample size limited statistical power to detect modest between-regimen differences, particularly across multiple biomarkers. Accordingly, the primary inference pertains to within-person change from baseline to w48, whereas between-regimen contrasts are secondary and exploratory. Second, all regimens evaluated were INSTI-based but differed in both the INSTI agent and the accompanying NRTI backbone; therefore, observed differences cannot be attributed to the INSTI component alone and should be interpreted as comparisons of complete regimens rather than head-to-head INSTI effects. Future studies including NNRTI- or PI-based regimens with similar NRTI components would be needed to more clearly disentangle drug class specific effects on immune and inflammatory biomarkers. Third, the non-randomized design may introduce selection bias and residual confounding from unmeasured factors despite multivariable adjustment. Fourth, baseline differences in several biomarkers indicate underlying heterogeneity at ART initiation, which may affect the interpretability of absolute between-group comparisons; this was addressed by focusing on within-person longitudinal changes and adjusting analyses for covariates that differed at baseline. Fifth, concomitant medications and lifestyle factors were not systematically captured and may have influenced biomarker trajectories; additionally, longer follow-up is needed to assess durability beyond w48. Sixth, sex-based analyses were not feasible due to baseline imbalance across groups, which is relevant given known sex-related differences in soluble mediators and HIV outcomes [53]. Seventh, use of archived biobank samples, although processed under standardized conditions, may introduce pre-analytical variability (e.g., storage duration and freeze-thaw effects). Eighth, missing data were handled using multiple imputation under a missing-at-random assumption, which cannot be formally verified and may introduce bias if violated. Ninth, only two time points were available, limiting the ability to characterize non-linear biomarker trajectories.

Although no formal adjustment for multiple comparisons was applied and p values are therefore nominal-raising the risk of type I error, particularly for between-regimen contrasts across the biomarker panel-these results should be interpreted cautiously and considered hypothesis-generating. In this context, the heterogeneity reported across studies of inflammatory biomarkers, likely driven by differences in populations, regimens and follow-up, further underscores the need for harmonized biomarker evaluation strategies [7].

## Conclusion

While all INSTIs-based regimens were associated with immunologic improvement, modest differences in immune activation and regulatory markers suggest each regimen may have a distinct immunomodulatory profile. The clinical relevance of these differences remains uncertain and warrants investigation in longer-term studies with robust clinical endpoints. Future research should examine the relationship between biomarker dynamics and the incidence of comorbidities to assess whether these immunologic variations translate into meaningful health outcomes for PLWH. Long-term monitoring of these biomarkers may help personalize ART and identify patients who could benefit from tailored strategies to reduce comorbidity risk.

## Supporting information

S1 Table Limits of detection for each biomarker. (PDF)

## References

[1] Chawla A, Wang C, Patton C, Murray M, Punekar Y, de Ruiter A, et al. A Review of Long-Term Toxicity of Antiretroviral Treatment Regimens and Implications for an Aging Population. Infect Dis Ther. 2018;7(2):183–95. doi: 10.1007/s40121-018-0201-6 29761330 PMC5986685

[2] Teeraananchai S, Kerr SJ, Amin J, Ruxrungtham K, Law MG. Life expectancy of HIV-positive people after starting combination antiretroviral therapy: a meta-analysis. HIV Med. 2017;18(4):256–66. doi: 10.1111/hiv.12421 27578404

[3] Gueler A, Moser A, Calmy A, Günthard HF, Bernasconi E, Furrer H, et al. Life expectancy in HIV-positive persons in Switzerland: matched comparison with general population. AIDS. 2017;31(3):427–36. doi: 10.1097/QAD.0000000000001335 27831953 PMC5302412

[4] Pandrea I, Landay A, Wilson C, Stock J, Tracy R, Apetrei C. Using the pathogenic and nonpathogenic nonhuman primate model for studying non-AIDS comorbidities. Curr HIV/AIDS Rep. 2015;12(1):54–67. doi: 10.1007/s11904-014-0245-5 25604236 PMC4369284

[5] Hsue PY, Deeks SG, Hunt PW. Immunologic basis of cardiovascular disease in HIV-infected adults. J Infect Dis. 2012;205 Suppl 3(Suppl 3):S375-82. doi: 10.1093/infdis/jis200 22577211 PMC3349295

[6] Deeks SG, Tracy R, Douek DC. Systemic effects of inflammation on health during chronic HIV infection. Immunity. 2013;39(4):633–45. doi: 10.1016/j.immuni.2013.10.001 24138880 PMC4012895

[7] Llibre JM, Cahn PE, Lo J, Barber TJ, Mussini C, van Welzen BJ, et al. Changes in Inflammatory and Atherogenesis Biomarkers With the 2-Drug Regimen Dolutegravir Plus Lamivudine in Antiretroviral Therapy-Experienced, Virologically Suppressed People With HIV-1: A Systematic Literature Review. Open Forum Infect Dis. 2022;9(4):ofac068. doi: 10.1093/ofid/ofac068 35265729 PMC8900931

[8] Furman D, Campisi J, Verdin E, Carrera-Bastos P, Targ S, Franceschi C, et al. Chronic inflammation in the etiology of disease across the life span. Nat Med. 2019;25(12):1822–32. doi: 10.1038/s41591-019-0675-0 31806905 PMC7147972

[9] Temu TM, Polyak SJ, Zifodya JS, Wanjalla CN, Koethe JR, Masyuko S, et al. Endothelial Dysfunction Is Related to Monocyte Activation in Antiretroviral-Treated People With HIV and HIV-Negative Adults in Kenya. Open Forum Infect Dis. 2020;7(10):ofaa425. doi: 10.1093/ofid/ofaa425 33094120 PMC7568437

[10] Klos B, Patel P, Rose C, Bush T, Conley L, Kojic EM, et al. Lower serum adiponectin level is associated with lipodystrophy among HIV-infected men in the Study to Understand the Natural History of HIV/AIDS in the Era of Effective Therapy (SUN) study. HIV Med. 2019;20(8):534–41. doi: 10.1111/hiv.12754 31149766

[11] Tenorio AR, Zheng Y, Bosch RJ, Krishnan S, Rodriguez B, Hunt PW, et al. Soluble markers of inflammation and coagulation but not T-cell activation predict non-AIDS-defining morbid events during suppressive antiretroviral treatment. J Infect Dis. 2014;210(8):1248–59. doi: 10.1093/infdis/jiu254 24795473 PMC4192039

[12] Qi Q, Hua S, Clish CB, Scott JM, Hanna DB, Wang T, et al. Plasma Tryptophan-Kynurenine Metabolites Are Altered in Human Immunodeficiency Virus Infection and Associated With Progression of Carotid Artery Atherosclerosis. Clin Infect Dis. 2018;67(2):235–42. doi: 10.1093/cid/ciy053 29415228 PMC6031054

[13] Dow DE, Bartlett JA. Dolutegravir, the Second-Generation of Integrase Strand Transfer Inhibitors (INSTIs) for the Treatment of HIV. Infect Dis Ther. 2014;3(2):83–102. doi: 10.1007/s40121-014-0029-7 25134686 PMC4269626

[14] Ambrosioni J, Levi L, Alagaratnam J, Van Bremen K, Mastrangelo A, Waalewijn H, et al. Major revision version 12.0 of the European AIDS Clinical Society guidelines 2023. HIV Med. 2023;24(11):1126–36. doi: 10.1111/hiv.13542 37849432

[15] Gandhi RT, Landovitz RJ, Sax PE, Smith DM, Springer SA, Günthard HF, et al. Antiretroviral Drugs for Treatment and Prevention of HIV in Adults: 2024 Recommendations of the International Antiviral Society-USA Panel. JAMA. 2025;333(7):609–28. doi: 10.1001/jama.2024.24543 39616604

[16] Pantazis N, Sabin CA, Grabar S, Van der Valk M, Jarrin I, van Sighem A, et al. Changes in bodyweight after initiating antiretroviral therapy close to HIV-1 seroconversion: an international cohort collaboration. Lancet HIV. 2024;11(10):e660–9. doi: 10.1016/S2352-3018(24)00183-8 39186940

[17] Gan L, Xie X, Fu Y, Yang X, Ma S, Kong L, et al. Comparison of dolutegravir+Lamivudine and bictegravir/emtricitabine/tenofovir alafenamide in antiretroviral therapy-naïve patients infected with HIV: preliminary results from clinical practice. Expert Rev Anti Infect Ther. 2024;22(10):877–84. doi: 10.1080/14787210.2023.2279719 37927079

[18] Savinelli S, Newman E, Mallon PWG. Metabolic Complications Associated with Use of Integrase Strand Transfer Inhibitors (InSTI) for the Treatment of HIV-1 Infection: Focus on Weight Changes, Lipids, Glucose and Bone Metabolism. Curr HIV/AIDS Rep. 2024;21(6):293–308. doi: 10.1007/s11904-024-00708-x 39207722 PMC11486773

[19] García-Ruiz de Morales AG, Suárez Robles M, Pérez-Elías MJ, Negredo E, Alcamí J, Gómez Rodríguez CE, et al. Metabolic Complications After Initiating BIC/FTC/TAF Versus DTG + 3TC in Antiretroviral-therapy-naive Adults With HIV: A Multicenter Prospective Cohort Study. Clin Infect Dis. 2025;81(2):263–70. doi: 10.1093/cid/ciae645 40155359

[20] Funderburg NT, Huang SSY, Cohen C, Ailstock K, Cummings M, Lee JC, et al. Changes to inflammatory markers during 5 years of viral suppression and during viral blips in people with HIV initiating different integrase inhibitor based regimens. Front Immunol. 2024;15:1488799. doi: 10.3389/fimmu.2024.1488799 39600696 PMC11590120

[21] Daar ES, Orkin C, Sax PE, Hagins D, Pozniak A, Workowski K, et al. Long-term metabolic changes with bictegravir/emtricitabine/tenofovir alafenamide or dolutegravir-containing regimens for HIV. AIDS Res Ther. 2025;22(1):45. doi: 10.1186/s12981-025-00732-w 40197415 PMC11978100

[22] Caro-Murillo AM, Castilla J, Pérez-Hoyos S, Miró JM, Podzamczer D, Rubio R, et al. Spanish cohort of naïve HIV-infected patients (CoRIS): rationale, organization and initial results. Enferm Infecc Microbiol Clin. 2007;25(1):23–31. doi: 10.1157/13096749 17261243

[23] Sobrino-Vegas P, Gutiérrez F, Berenguer J, Labarga P, García F, Alejos-Ferreras B, et al. The Cohort of the Spanish HIV Research Network (CoRIS) and its associated biobank; organizational issues, main findings and losses to follow-up. Enferm Infecc Microbiol Clin. 2011;29(9):645–53. doi: 10.1016/j.eimc.2011.06.002 21820763

[24] García-Merino I, de Las Cuevas N, Jiménez JL, Gallego J, Gómez C, Prieto C, et al. The Spanish HIV BioBank: a model of cooperative HIV research. Retrovirology. 2009;6:27. doi: 10.1186/1742-4690-6-27 19272145 PMC2667474

[25] Mokoena H, Hanser S, Mabhida SE, Choshi J, Sekgala MD, Nkambule BB, et al. The Pathological Link Between Elevated Markers of Inflammation, Endothelial Activation, and Cardiovascular Diseases in People Living with HIV on Combination Antiretroviral Therapy: A Systematic Review. J Inflamm Res. 2025;18:17197–210. doi: 10.2147/JIR.S472896 41399534 PMC12702278

[26] Obare LM, Stephens VR, Wanjalla CN. Understanding residual risk of cardiovascular disease in people with HIV. Curr Opin HIV AIDS. 2025;20(4):319–30. doi: 10.1097/COH.0000000000000953 40397567 PMC12765488

[27] Caetano DG, Ribeiro-Alves M, Hottz ED, Vilela LM, Cardoso SW, Hoagland B, et al. Increased biomarkers of cardiovascular risk in HIV-1 viremic controllers and low persistent inflammation in elite controllers and art-suppressed individuals. Sci Rep. 2022;12(1):6569. doi: 10.1038/s41598-022-10330-9 35449171 PMC9023525

[28] Espiau M, Yeste D, Noguera-Julian A, Soler-Palacín P, Fortuny C, Ferrer R, et al. Adiponectin, Leptin and Inflammatory Markers in HIV-associated Metabolic Syndrome in Children and Adolescents. Pediatr Infect Dis J. 2017;36(2):e31–7. doi: 10.1097/INF.0000000000001394 27832021

[29] Hanser S, Mphekgwana PM, Moraba MM, Erasmus L, van Staden M. Increased endothelial biomarkers are associated with HIV antiretroviral therapy and C-reactive protein among a African rural population in Limpopo Province, South Africa. Front Public Health. 2022;10:980754. doi: 10.3389/fpubh.2022.980754 36407976 PMC9672841

[30] Sultana S, Elengickal A, Bensreti H, Belin de Chantemèle E, McGee-Lawrence ME, Hamrick MW. The kynurenine pathway in HIV, frailty and inflammaging. Front Immunol. 2023;14:1244622. doi: 10.3389/fimmu.2023.1244622 37744363 PMC10514395

[31] Buuren S van, Groothuis-Oudshoorn K. mice: Multivariate Imputation by Chained Equations inR. J Stat Soft. 2011;45(3). doi: 10.18637/jss.v045.i03

[32] R Core Team. R: A language and environment for statistical computing. Vienna, Austria: R Foundation for Statistical Computing. 2021.

[33] Wickham H, Vaughan D, Girlich M. Tidyr: Tidy messy data. 2024.

[34] Wickham H, François R, Henry L, Müller K, Vaughan D. dplyr: A Grammar of Data Manipulation. 2023.

[35] Wickham H. ggplot2: Elegant Graphics for Data Analysis. New York: Springer-Verlag. 2016.

[36] Bates D, Mächler M, Bolker B, Walker S. Fitting linear mixed-effects models using lme4. J Stat Softw. 2015;67(1):1–48.

[37] Routy J-P, Mehraj V, Vyboh K, Cao W, Kema I, Jenabian M-A. Clinical Relevance of Kynurenine Pathway in HIV/AIDS: An Immune Checkpoint at the Crossroads of Metabolism and Inflammation. AIDS Rev. 2015;17(2):96–106. 26035167

[38] Bailón L, Puertas MC, García-Guerrero MC, Moraes-Cardoso I, Aparicio E, Alarcón-Soto Y, et al. Impact of Dolutegravir Plus Lamivudine as First-line Antiretroviral Treatment on the Human Immunodeficiency Virus Type 1 Reservoir and Inflammatory Markers in Peripheral Blood. J Infect Dis. 2025;231(3):600–10. doi: 10.1093/infdis/jiae530 39465671 PMC11911789

[39] Calza L, Bon I, Pensalfine G, Vitale S, Appolloni L, Viale P. Changes in Serum Inflammatory Markers in Antiretroviral Therapy-Naive HIV-Infected Patients Starting Dolutegravir/Lamivudine or Tenofovir Alafenamide/Emtricitabine/Bictegravir. J Acquir Immune Defic Syndr. 2022;91(4):e9–11. doi: 10.1097/QAI.0000000000003077 35981265

[40] Suárez-García I, Alejos B, Hernando V, Viñuela L, Vera García M, Rial-Crestelo D, et al. Effectiveness and tolerability of dolutegravir/lamivudine for the treatment of HIV-1 infection in clinical practice. J Antimicrob Chemother. 2023;78(6):1423–32. doi: 10.1093/jac/dkad102 37099559 PMC10232237

[41] Gallant J, Lazzarin A, Mills A, Orkin C, Podzamczer D, Tebas P, et al. Bictegravir, emtricitabine, and tenofovir alafenamide versus dolutegravir, abacavir, and lamivudine for initial treatment of HIV-1 infection (GS-US-380-1489): a double-blind, multicentre, phase 3, randomised controlled non-inferiority trial. Lancet. 2017;390(10107):2063–72. doi: 10.1016/S0140-6736(17)32299-7 28867497

[42] Yang J, Wang L, Zhang X, Liu L, Shen Y, Qi T, et al. Safety and efficacy of lamivudine/dolutegravir vs. bictegravir/emtricitabine/tenofovir alafenamide in antiretroviral-naive adults with HIV-1 infection in Shanghai, China: a single-centre retrospective study. J Med Microbiol. 2025;74(1):001949. doi: 10.1099/jmm.0.001949 39773780 PMC12451750

[43] Quiros-Roldan E, Castelli F, Bonito A, Vezzoli M, Calza S, Biasiotto G, et al. The impact of integrase inhibitor-based regimens on markers of inflammation among HIV naïve patients. Cytokine. 2020;126:154884. doi: 10.1016/j.cyto.2019.154884 31670006

[44] Martínez-Sanz J, Ron R, Moreno E, Sánchez-Conde M, Muriel A, López Cortés LF, et al. Similar CD4/CD8 Ratio Recovery After Initiation of Dolutegravir Plus Lamivudine Versus Dolutegravir or Bictegravir-Based Three-Drug Regimens in Naive Adults With HIV. Front Immunol. 2022;13:873408. doi: 10.3389/fimmu.2022.873408 35432298 PMC9009371

[45] Iyer SS, Cheng G. Role of interleukin 10 transcriptional regulation in inflammation and autoimmune disease. Crit Rev Immunol. 2012;32(1):23–63. doi: 10.1615/critrevimmunol.v32.i1.30 22428854 PMC3410706

[46] Jianu C, Itu-Mureşan C, Drugan C, Filipescu I, Topan AV, Jianu ME, et al. Evaluation of several serum interleukins as markers for treatment effectiveness in naïve HIV infected patients: A pilot study. PLoS One. 2021;16(11):e0260007. doi: 10.1371/journal.pone.0260007 34784398 PMC8594820

[47] Plevriti A, Lamprou M, Mourkogianni E, Skoulas N, Giannakopoulou M, Sajib MS, et al. The Role of Soluble CD163 (sCD163) in Human Physiology and Pathophysiology. Cells. 2024;13(20):1679. doi: 10.3390/cells13201679 39451197 PMC11506427

[48] Kneidl J, Mysore V, Geraci J, Tuchscherr L, Löffler B, Holzinger D, et al. Soluble CD163 masks fibronectin-binding protein A-mediated inflammatory activation of Staphylococcus aureus infected monocytes. Cell Microbiol. 2014;16(3):364–77. doi: 10.1111/cmi.12225 24118665

[49] Alvarado-Vazquez PA, Bernal L, Paige CA, Grosick RL, Moracho Vilrriales C, Ferreira DW, et al. Macrophage-specific nanotechnology-driven CD163 overexpression in human macrophages results in an M2 phenotype under inflammatory conditions. Immunobiology. 2017;222(8–9):900–12. doi: 10.1016/j.imbio.2017.05.011 28545809 PMC5718187

[50] Beltrán LM, Muñoz Hernández R, de Pablo Bernal RS, García Morillo JS, Egido J, Noval ML, et al. Reduced sTWEAK and increased sCD163 levels in HIV-infected patients: modulation by antiretroviral treatment, HIV replication and HCV co-infection. PLoS One. 2014;9(3):e90541. doi: 10.1371/journal.pone.0090541 24594990 PMC3942443

[51] Chen S, Saeed AFUH, Liu Q, Jiang Q, Xu H, Xiao GG, et al. Macrophages in immunoregulation and therapeutics. Signal Transduct Target Ther. 2023;8(1):207. doi: 10.1038/s41392-023-01452-1 37211559 PMC10200802

[52] Guo L, Akahori H, Harari E, Smith SL, Polavarapu R, Karmali V, et al. CD163+ macrophages promote angiogenesis and vascular permeability accompanied by inflammation in atherosclerosis. J Clin Invest. 2018;128(3):1106–24. doi: 10.1172/JCI93025 29457790 PMC5824873

[53] Krebs SJ, Slike BM, Sithinamsuwan P, Allen IE, Chalermchai T, Tipsuk S, et al. Sex differences in soluble markers vary before and after the initiation of antiretroviral therapy in chronically HIV-infected individuals. AIDS. 2016;30(10):1533–42. doi: 10.1097/QAD.0000000000001096 26990631 PMC4889571

