# Correction: Longitudinal changes in circulating biomarkers from baseline to week 48 in treatment-Naïve people living with HIV initiating integrase inhibitor-based antiretroviral therapy

**DOI:** 10.1371/journal.pone.0354685

**Published:** 2026-07-24

**Authors:** Jose-Ramon Blanco, Miguel Torralba, María Saumoy, Antonia Alcaraz, Mariano Matarranz del Amo, Julian Olalla, Fernando Dronda, Nisa Boukichou-Abdelkader, Enrique Bernal, Sergio Padilla, Joaquim Peraire, Francisca Artigues, María-Jesús Bustinduy-Odriozola, Helena Albendin Iglesias, Alberto Delgado, Arkaitz Imaz, Laura Pérez-Martinez, CoRIS Cohort

The 18^th^ author’s name is spelled incorrectly. The correct name is: CoRIS cohort.
