# Supplementary material for: Longitudinal changes in circulating biomarkers from baseline to week 48 in treatment-Naïve people living with HIV initiating integrase inhibitor-based antiretroviral therapy
Source: PLoS One. 2026 Feb 18;21(2):e0343230. doi: 10.1371/journal.pone.0343230 (PMC12915926; doi:10.1371/journal.pone.0343230)
Supplement: S1 Table — (PDF) [file pone.0343230.s001.pdf]

Supplementary Table S1. Assay methods and minimum detectable dose (MDD) for circulating biomarkers.

Biomarker	Procedure	Minimum Detectable Dose (MDD)	Commercial
CD14	Luminex assays	0.0396 ng/ml	Bio-Techne
CD40	Luminex assays	0.0347 ng/ml	Bio-Techne
CD163	Luminex assays	0.53 ng/ml	Bio-Techne
LBP	Luminex assays	0.0839 ng/ml	Bio-Techne
TNF	Luminex assays	0.0012 ng/ml	Bio-Techne
ICAM-1	Luminex assays	0.0879 ng/ml	Bio-Techne
IL-6	Luminex assays	0.0017 ng/ml	Bio-Techne
IL-8	Luminex assays	0.0018 ng/ml	Bio-Techne
IL-10	Luminex assays	0.0016 ng/ml	Bio-Techne
E-selectin	Luminex assays	0.0188 ng/ml	Bio-Techne
P-selectin	Luminex assays	0.009 ng/ml	Bio-Techne
VCAM-1	Luminex assays	0.238 ng/ml	Bio-Techne
Adiponectin	Luminex assays	0.148 ng/ml	Bio-Techne
Soluble P-selectin	Quantikine® ELISA	0,20 ng/ml	Invitrogen
CRP	Quantikine® ELISA	10 ng/ml	Invitrogen
Leptin	Quantikine® ELISA	0,0078 ng/ml	R&D Systems
Tryptophan	Quantikine® ELISA	0,55 ng/ml	MyBioSource
Kynurenic acid	Quantikine® ELISA	0,89 ng/ml	MyBioSource
Quinolinic acid	Quantikine® ELISA	0,60 ng/ml	MyBioSource

Abbreviations: CD14, cluster of differentiation 14; CD40, cluster of differentiation 40; CD163, cluster of differentiation 163; LBP, lipopolysaccharide-binding protein; TNF, tumor necrosis factor; ICAM-1, intercellular adhesion molecule; IL, interleukin; VCAM-1, vascular cell adhesion molecule 1; CRP, C-reactive protein.
